# Connecting Biochemical Photosynthesis Models with Crop Models to Support Crop Improvement

**DOI:** 10.3389/fpls.2016.01518

**Published:** 2016-10-13

**Authors:** Alex Wu, Youhong Song, Erik J. van Oosterom, Graeme L. Hammer

**Affiliations:** ^1^Centre for Plant Science, Queensland Alliance for Agriculture and Food Innovation, The University of QueenslandBrisbane, QLD, Australia; ^2^ARC Centre of Excellence for Translational Photosynthesis, The University of QueenslandBrisbane, QLD, Australia

**Keywords:** biochemical photosynthesis model, photosynthesis light response, RUE, canopy photosynthesis, crop model, crop improvement, cross-scale modeling, specific leaf nitrogen

## Abstract

The next advance in field crop productivity will likely need to come from improving crop use efficiency of resources (e.g., light, water, and nitrogen), aspects of which are closely linked with overall crop photosynthetic efficiency. Progress in genetic manipulation of photosynthesis is confounded by uncertainties of consequences at crop level because of difficulties connecting across scales. Crop growth and development simulation models that integrate across biological levels of organization and use a gene-to-phenotype modeling approach may present a way forward. There has been a long history of development of crop models capable of simulating dynamics of crop physiological attributes. Many crop models incorporate canopy photosynthesis (source) as a key driver for crop growth, while others derive crop growth from the balance between source- and sink-limitations. Modeling leaf photosynthesis has progressed from empirical modeling via light response curves to a more mechanistic basis, having clearer links to the underlying biochemical processes of photosynthesis. Cross-scale modeling that connects models at the biochemical and crop levels and utilizes developments in upscaling leaf-level models to canopy models has the potential to bridge the gap between photosynthetic manipulation at the biochemical level and its consequences on crop productivity. Here we review approaches to this emerging cross-scale modeling framework and reinforce the need for connections across levels of modeling. Further, we propose strategies for connecting biochemical models of photosynthesis into the cross-scale modeling framework to support crop improvement through photosynthetic manipulation.

## Introduction

Global crop production needs to approximately double by 2050 to meet the projected demands from rising population, diet shifts, and increasing needs for biofuels. Current trends in yield improvement of major field crops (i.e., wheat, rice, maize, and soybean), however, are insufficient to meet the projected demand (Ray et al., 2013; Fischer et al., 2014). In addition, more frequent extreme weather conditions associated with climate change are likely to have negative impacts on global crop yields (Lobell and Gourdji, 2012). Yield improvement in the past 50 years has been achieved through improved genetics via conventional plant breeding coupled with enhanced agronomy and crop protection (e.g., maize in the US; Duvick, 2005). Crop yield can be viewed as the product of resource capture (e.g., light, water, and nitrogen), the efficiency with which these resources are converted into biomass, and the extent of partitioning of biomass to harvestable product (i.e., harvest index). Yield improvement to date has been largely associated with improved efficiency to capture resources and with harvest index (Tollenaar and Lee, 2006; Fischer, 2007). While opportunities remain for improving grain yield through improved resource capture (e.g., root architecture Singh et al., 2012) and biomass partitioning (Duvick, 2005; Messina et al., 2009), these traits may be approaching their biological limits (Duvick and Cassman, 1999; Long et al., 2015). The one area in which there is little evidence of improvement is in crop use efficiency of resources, aspects of which depend on overall crop photosynthetic efficiency. While there is evidence of genetic variation in resource use efficiency (Henderson et al., 1998; Hammer et al., 2010), causal links to yield improvement are limited (Fischer et al., 1998; Sadras and Lawson, 2011).

Enhancing photosynthesis is becoming one focus for pursuing greater crop use efficiency of resources (Long et al., 2006; Zhu et al., 2010). This is made possible by: (1) our understanding of the photosynthetic pathway, (2) emergence of high-performance computing for simulating photosynthetic processes across scales from photosynthetic biochemistry to crop level, and (3) advances in genetic engineering (Long et al., 2015). There is now a well-defined agenda for the genetic manipulation of the biochemical pathway of photosynthesis at the leaf level for crop yield improvement (see review of Evans, 2013). Examples include engineering the C_4_ photosynthetic pathway into rice (http://c4rice.irri.org/) and improving leaf CO_2_ capture efficiency and light energy capture efficiency (http://photosynthesis.org.au). However, manipulation of photosynthesis at the biochemical level may not necessarily correlate with crop yield (see reviews of Sinclair et al., 2004; Long et al., 2006). This is because the gap between the biochemical and crop level of biological organizations confounds crop improvement (Sinclair et al., 2004; Hammer et al., 2006). Integrating to crop level is complicated by the (photosynthetic) genetic controls, dynamics of crop growth and development and their interactions with the environment.

**Figure 1:**
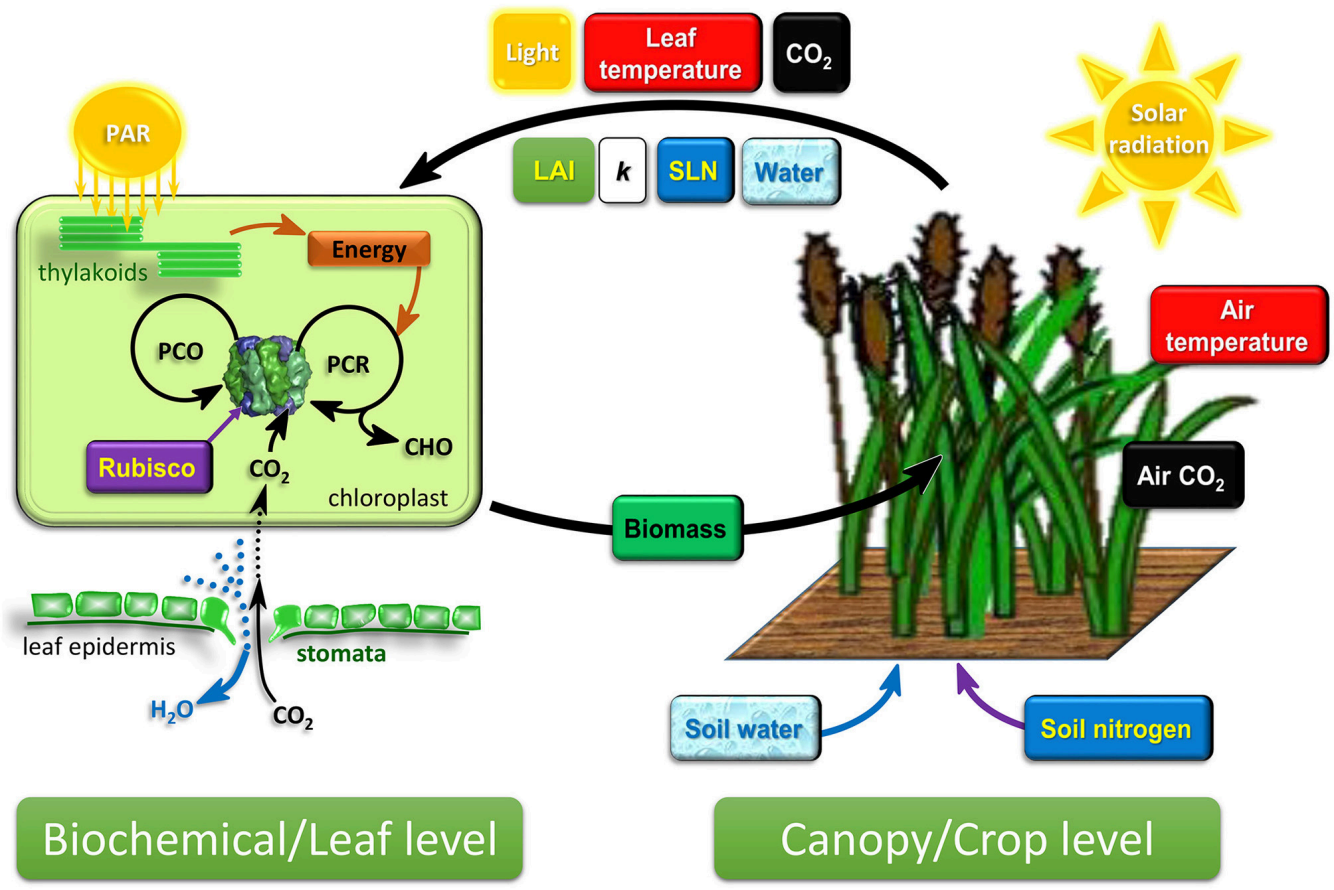
**Schematic diagram of the emerging cross-scale modeling framework connecting biochemical/leaf-level photosynthesis and canopy/crop-level growth and development dynamics**. Crop growth and development is driven by the development of canopy leaf area and canopy biomass growth, both of which are influenced by the prevailing environment and the photosynthesis output of individual leaves in the canopy. The canopy captures resources from the environment. Leaf photosynthesis is driven by the attributes of the crop canopy and leaves. LAI, SLN, and crop water status are determined by crop scale growth and development dynamics, while light, leaf temperature, and CO_2_ experienced by leaves are influenced by canopy attributes, LAI and *k*. This two-way connection between biochemical and crop level (the two thick arrows) is an important consideration in the cross-scale modeling framework. PAR, photosynthetic active radiation; LAI, leaf area index; *k*, canopy light extinction coefficient; SLN, specific leaf nitrogen; PCR, photosynthetic carbon reduction cycle; PCO, photorespiratory carbon oxidation cycle; CHO, carbohydrates synthesized by photosynthesis.

There is a need to close the gap between the biochemical and crop levels using modeling to help accelerate progress in photosynthesis enhancement for crop improvement. A cross-scale modeling approach connecting these two levels with each other and with environmental effects provides a valuable theoretical framework for closing this gap. Crop level growth and development dynamics and effects of environments can be simulated with crop models that incorporate both source- and sink-limited crop growth (Hammer et al., 2010; Gent and Seginer, 2012; Fatichi et al., 2014). Nonetheless, canopy photosynthesis is a key driver in crop models. Photosynthesis models, focused at different levels of modeling, have evolved from empirical modeling of the photosynthetic light response (Blackman, 1905) to upscaling to the canopy level (Monsi and Saeki, 1953), and to connections with crop models (e.g., de Wit et al., 1978). At the crop level, canopy Radiation Use Efficiency (RUE) has been used successfully to determine the sum of photosynthetic output of individual leaves in the canopy (Monteith and Moss, 1977; Sinclair and Muchow, 1999) and RUE underpins crop growth prediction in many crop models (Parent and Tardieu, 2014). This simple approach avoids the need to connect photosynthesis between biochemical and canopy levels, although theoretical derivations have shown the clear connection of RUE with leaf photosynthesis within crop canopies (Hammer and Wright, 1994). These empirical canopy photosynthesis modeling approaches have been useful, but lack the biological functionality to capture canopy level consequences of genetic modification of photosynthesis at the biochemical level attributed to their aggregated nature. Biochemical models of photosynthesis, based on key biochemical processes of photosynthesis, have been developed at the leaf level (Farquhar et al., 1980; von Caemmerer and Farquhar, 1981; Farquhar and von Caemmerer, 1982; von Caemmerer and Furbank, 1999; von Caemmerer, 2000). These more mechanistic biochemical photosynthesis modeling approaches have been useful in interpreting gas exchange measurements of steady-state CO_2_ assimilation of leaves and in predicting responses of leaf photosynthesis to genetic and environmental controls of photosynthesis and have been subsequently upscaled to the canopy level (Sellers et al., 1992; Leuning et al., 1995; de Pury and Farquhar, 1997). However, the biochemical models, by their intrinsic instantaneous nature, lack the integrative ability to capture interactions with key aspects of crop growth and development dynamics throughout the crop life cycle. Cross-scale modeling that connects across scales of biological organization and utilizes model developments in both photosynthesis and crop growth and development dynamics provides a means to capture the dynamics of photosynthesis manipulation to support crop improvement. In this review we pursue three objectives to aid the development of cross-scale modeling. These are to:

1. Summarize the emerging cross-scale modeling framework for connecting photosynthesis models at canopy and biochemical levels (Figure 1);
2. Identify avenues to improve connections in the cross-scale modeling framework with effects of environmental factors and crop physiological attributes;
3. Propose strategies for connecting biochemical photosynthesis models into the cross-scale modeling framework.

## Cross-scale modeling framework for connecting photosynthesis models at canopy and biochemical levels

In crop models, canopy photosynthesis is a key driver of crop growth (de Wit, 1965; Duncan et al., 1967; Goudriaan and van laar, 1978; Thornley, 2002; El-Sharkawy, 2011). Early canopy photosynthesis modeling involved either (i) integrating photosynthesis of individual leaves in the canopy (de Wit, 1965; Duncan et al., 1967; Goudriaan and van laar, 1978), or (ii) utilizing the simple linear relationship observed between accumulated crop canopy biomass and intercepted solar radiation (Shibles and Weber, 1965, 1966; Williams et al., 1965; Monteith and Moss, 1977), also known as RUE (g/MJ) (Sinclair and Muchow, 1999). Depending on the details required for simulating canopy photosynthesis, either type of model can be used to drive growth in crop models. For example, crop models such as GECROS (Yin and van Laar, 2005) use the first approach, while APSIM (Holzworth et al., 2014) and DSSAT (Jones et al., 2003) use the second approach, although APSIM does have the provision to switch between the two approaches by incorporating an optional module with leaf photosynthesis light response curves and canopy architecture algorithms (Hammer et al., 2009).

### Leaf photosynthesis light response modeling

Radiation (or light) intensity (*I*) is the key environmental factor for photosynthesis. Hence, the explicit modeling of the response of leaf photosynthesis (*A*) to *I*, or the *A*/*I* curve, has been a key focus since the beginning of leaf photosynthesis modeling. Modeling of leaf *A*/*I* curves, referred to as photosynthetic light-response (PLR) modeling, has a long history. Blackman (1905) describes a response of photosynthesis to light that increases linearly with *I* with a slope α (maximum quantum yield) until the maximum rate of photosynthesis (*A*_max_), where CO_2_ supply becomes limiting (Table 1). α is the maximum efficiency with which light can be converted to chemical energy by photosynthesis (assimilated CO_2_ per quantum of absorbed light). It is known to be similar across a wide range of C_3_ species when expressed on the basis of absorbed light (Ogren, 1993) and does not depend on either irradiance or leaf nitrogen content per unit leaf area (Thornley, 1998). The two parts of the photosynthesis light response curve are described as light- and CO_2_-limited, respectively. This approach is plausible, but is restricted by the sharp discontinuity at the transition between the two limiting factors.

**Table 1:** **List of major photosynthetic light-response models of leaf photosynthesis (***A***) response to light intensity (***I***)**.

**Model type**	**Generic function**	**Parameter definition**	**References**
Linear model	$A=\{\begin{array}{c} \alpha I,I\leq A_{\text{max}}/\alpha \\ A_{\text{max}},I>A_{\text{max}}/\alpha \end{array}$	*A*_max_, maximum rate of photosynthesis; *I*, light intensity; *α*, maximum quantum yield.	Blackman, 1905
Rectangular hyperbola	$A=\frac{aIA_{\text{max}}}{aI\;+\;A_{\text{max}}}$	Same as the linear model.	Maskell, 1928
Non-rectangular hyperbola	$\theta A^{2}-\left(\alpha I+A_{\text{max}}\right)A+\alpha IA_{\text{max}}=0$	θ, empirical convexity factor for the *A*/*I* curve; others, same as the linear model.	Thornley, 1976
Exponential equation	$A=A_{\text{max}}[1-e^{-\alpha I/A_{\text{max}}}]$	Same as the linear model.	Hammer and Wright, 1994
Modified rectangular hyperbola	$A=\delta \frac{1\;-\;\beta I}{1\;+\;\gamma I}\left(I-I_{\text{c}}\right)$	*δ, β*, and γ are empirical coefficients; *I*c, compensation irradiance; others, some as the linear model.	Ye, 2007

To overcome this issue, responses using rectangular hyperbola type functions (Table 1) were developed (Maskell, 1928). This type of function, based on Michaelis and Menten's equation, reproduces the curvilinear trend of *A*/*I* curves. However, it poorly describes *A*/*I* curves at saturating levels of CO_2_ and overestimates *A*_max_, α and dark respiration (*R*_d_), because the limitation on photosynthesis of the physical diffusion of CO_2_ from the atmosphere to the site of carboxylation in leaves has not been taken into account (Akhkha, 2010).

Rabinowitch (1951) combined a simplified description of biochemical reactions of photosynthesis and the physical diffusion of CO_2_ and generated the non-rectangular hyperbola type function (Table 1). Modeling of *A*/*I* curve using this function type has since been pursued by many authors with numerous variations (Ogren, 1993; Thornley, 1998, 2002; Xu et al., 2014). In addition to *A*_max_ and α, the non-rectangular hyperbola type function has an additional parameter θ (the curvature factor), which governs the “convexity” of the *A*/*I* curve. Ogren (1993) concluded that the *A*/*I* curve is usually truncated at the transition from electron-transport limitation to a limitation by ribulose-1,5-bisphosphate carboxylase-oxygenase (Rubisco); the convexity changes with CO_2_ levels as the transition shifts, with respect to *I*, in the *A*/*I* curve. Marshall and Biscoe (1980) subsequently extended the function to include *R*_d_. The rectangular hyperbola function is a special case of the non-rectangular hyperbola function (Thornley, 1998). Exponential functions (Table 1) have also been used to model *A*/*I* curves of C_3_ and C_4_ species based on experimental observations (Hammer and Wright, 1994).

Ye (2007) developed a modified rectangular hyperbola function with empirical coefficients (Table 1), which has improved fit to *A*/*I* curves of *Oryza sativa*. The derivative of the modified rectangular hyperbola (at *I* = 0) gives the maximum quantum yield. In subsequent work, Ye et al. (2013) developed a mechanistic *A*/*I* model based on the modified rectangular hyperbola function and ascribed the then empirical coefficients to light-harvesting characteristics and associated biophysical parameters of photosynthetic pigment molecule—mechanisms that underpin photosynthetic electron transport via photosystem II. This model is applicable for both C_3_ and C_4_ species, and demonstrated excellent levels of correspondence with observed responses (Ye et al., 2013).

Besides the key environmental factor of light, physiological attributes of the plants have significant effects on photosynthesis (Evans, 1989; Sinclair and Horie, 1989). Hence, their incorporation in PLR models is important. The PLR models have also evolved to incorporate improved connections between model parameters and crop physiological attributes that influence photosynthesis. For example, *A*_max_, a parameter present in almost all PLR models, has been related to leaf nitrogen content per unit leaf area, which is commonly referred to as specific leaf nitrogen (SLN) (Muchow and Sinclair, 1994). Use of advanced PLR models with links between model parameters and crop physiological attributes will contribute to improving accuracy and connections in the cross-scale modeling.

### Canopy light distribution modeling

In the crop production environment, incident solar radiation is dynamic and can be described with solar geometry models and atmospheric transmissivity coefficients (Campbell, 1977; Brock, 1981; Hammer and Wright, 1994; Monteith and Unsworth, 2013a). At the earth's surface, solar radiation can be separated into direct and diffuse components, with the latter due to scattering of light as it travels through the atmosphere. Both components of incident light are important for canopy photosynthesis (de Wit, 1959, 1965; Duncan et al., 1967) and have become an essential part of canopy photosynthesis modeling (Duncan et al., 1967; Hammer and Wright, 1994; de Pury and Farquhar, 1997).

The complexity of canopy photosynthesis was first described by Boysen Jensen (1932), who demonstrated that canopy photosynthesis light response differs from that of isolated leaves in the canopy (Hirose, 2005). It differs because leaves in a canopy are exposed to different light environments throughout the day, depending on their spatial arrangement (e.g., leaf position in the canopy and leaf angle), the solar radiation intensity, and the location of the sun as it crosses the sky on its diurnal and seasonal course. In general, top leaf layers in the canopy receive more direct and intense light, while lower leaf layers receive much less light due to shading, although some direct light penetrates even to the lowest layer. This heterogeneity in radiation is a main factor complicating canopy photosynthesis.

Monsi and Saeki (1953) were among the first to quantify how sunlight is diminished as it proceeds into a canopy and is intercepted by leaves. They developed the first canopy light distribution model to characterize the light environment in a canopy. This model can be classified as a 1D canopy model, where the light environment only varies vertically in canopies and is assumed homogenous in the horizontal plane. Monsi and Saeki (1953) showed that light attenuation is approximately exponential in canopies and can thus be modeled by the common form of the Beer-Lambert equation for light extinction in homogenous media (in the context of a canopy):

(1) $$\begin{array}{l} I=I_{0}e^{-k*LAI} \end{array}$$

where *LAI* is the cumulative leaf area index from the top of the canopy above the layer of interest, *I* is the light intensity received at the layer of interest, *I*_0_ is the light intensity at the top of the canopy, and *k* is the light extinction coefficient. Equation (1) has been applied in many crop models that apply a static value of *k* for the canopy as spatial arrangement of leaves can be assumed homogeneous throughout the growing season. This simplification has led to wide adoption of Monsi and Saeki's approach in subsequent canopy photosynthesis modeling (Monsi and Saeki, 1953; Saeki, 1960; de Wit, 1965; Duncan et al., 1967) and it remained important for canopy light distribution modeling in later work (Sinclair et al., 1976; Hammer and Wright, 1994; Leuning et al., 1995; de Pury and Farquhar, 1997). Estimates of diurnal photosynthesis and growth of field crops using the 1D formalisms applied over a diverse range of environments have shown this approach to be effective and robust (Hammer and Wright, 1994; Hammer et al., 2009).

Canopy light distribution modeling has subsequently been expanded to include more detailed canopy models. Multi-layer models (de Wit, 1965; Monteith, 1965b; Duncan et al., 1967; Goudriaan, 1977) were developed by dividing the canopy into layers that are specified by their respective LAI. For each layer, LAI can be divided into sunlit and shaded leaf fractions and the sunlit fraction can be further divided into leaf angle classes (Duncan et al., 1967; Sinclair et al., 1976). The intensity of solar radiation reaching each fraction is calculated using the Beer-Lambert equation (Equation 1). The shaded fraction is assumed to intercept diffuse solar radiation and radiation reflected and transmitted through canopy layers. The sunlit fraction is assumed to intercept direct solar radiation in addition to the three types of radiation intercepted by the shaded fraction (Duncan et al., 1967; Leuning et al., 1995; de Pury and Farquhar, 1997). Later canopy models (e.g., Leuning et al., 1995; de Pury and Farquhar, 1997) have incorporated details about attenuation of different types of radiation through the use of different *k*-values. Simpler approximations for the multiple leaf angle classes, such as the three leaf-angle classes treatment (Goudriaan, 1988), or the spherical leaf angle distribution (de Wit et al., 1978), can be assumed to avoid the need for parameterizing leaf angles in the canopy. The spherical leaf angle distribution is a simple and robust approach for canopy photosynthesis and crop RUE modeling that has been widely implemented in later canopy photosynthesis models (Goudriaan, 1977; Hammer and Wright, 1994; Leuning et al., 1995; de Pury and Farquhar, 1997). Further, the multi-layer feature can be eliminated by integrating the canopy across LAI for both sunlit and shaded leaf fractions. This results in a simpler representation of the canopy that retains the distinction between sunlit and shaded leaf fractions, a necessity for capturing heterogeneous radiation in canopies. It has been shown that simulations using this single layer sun-shade modeling approach agree very closely with those using the multi-layer modeling approach in predicting canopy photosynthesis (de Pury and Farquhar, 1997) and agreed (within 5%) with results using a static 3D plant architecture model (Roupsard et al., 2008). This validates the robustness of the sun-shade modeling approach.

However, there is evidence that variation in *k* can have significant effects on crop growth. *k* can be influenced by developmental stage, canopy configurations (Evers et al., 2009) and canopy architectural traits, such as leaf shape, leaf angle, and internode length (Hirose, 2005; Kahlen et al., 2008). This requires a more dynamic *k* than used in Equation (1). Duncan et al. (1967) developed a model of *k* as a function of canopy average leaf angle and sun angle that could predict seasonal maize dry matter production with high accuracy (*r* = 0.94). Hammer et al. (2009) applied this approach in their modeling work and found that canopy-average leaf angle would only have significant effect on maize grain yield under very high yielding situations [well-watered and high (>6 plants/m^2^) planting density]. *k* has also been modeled empirically with respect to canopy LAI (Tahiri et al., 2006). The APSIM wheat model has the provision to specify *k* in terms of crop row spacing. Modeling of *k* has progressed from assuming a static value to connection with crop canopy attributes.

Despite its effectiveness and robustness, it can be argued that the 1D modeling approaches are limited in their ability to explicitly describe effects of canopy architectural traits on canopy light distribution. Emergence of 3D plant architecture models may overcome this limitation (Vos et al., 2010). This includes the RATP model (Sinoquet et al., 2001), which has the capacity to simulate the spatial distribution of light interception in an isolated tree crown using a static 3D canopy architecture model and a 3D extension of the Beer-Lambert equation (Equation 1). Further, Chen et al. (2014) developed a detailed 3D canopy architecture model for a tomato crop, which allows simulation of its growth and its effects on the canopy light distribution and absorption. The derived *k* using this model is significantly affected by leaf angle and internode length; *k* is higher during the early growth period (<30 days after first leaf appearance in this case), but stabilizes at a lower value for the remainder of crop growth. In this case, the early growth period contributed to less than 10% of the final shoot dry mass. Because biomass accumulation of field crops during these early developmental stages is generally only a small proportion of the biomass accumulated at maturity, the simpler 1D modeling approach, where *k* is assumed static throughout crop development, is generally effective and robust. However, the drawback is that it is more difficult to predict canopy light distribution if architectural traits (Song et al., 2013; Chen et al., 2014) or planting configuration (Evers et al., 2009) are manipulated. This is where the 3D approach may have some advantages.

### Connecting photosynthetic light-response models to canopy light distribution

One of the early approaches for modeling of canopy photosynthesis was based on combining the development in PLR models with the simplified canopy models for canopy light distribution calculation (de Wit, 1965; Duncan et al., 1967; Goudriaan and van laar, 1978). However, incorporation of this approach into crop models quickly illustrated that aspects of canopy photosynthesis are influenced by crop growth and development dynamics and *vice versa*. For example, many crop models generate canopy LAI over the crop growing season; this influences the amount of solar radiation intercepted by crop canopy, which in turn influences canopy photosynthesis and drives crop growth (Figure 1). The same basic modeling precept has been applied in more detailed canopy architectural models (Vos et al., 2010). Instead of using a simplified description of canopy structure (such as *k* and canopy LAI), those models explicitly describe the development of the 3D canopy architecture of plants (e.g., Chen et al., 2014). This can be coupled with Monte Carlo ray tracing for canopy light absorption calculation, which can be used to drive PLR models (Vos et al., 2010). Consideration of the two-way connection between the PLR models [which have advanced to become more accurate (Section Leaf Photosynthesis Light Response Modeling)] with canopy light distribution provides a foundation for canopy photosynthesis modeling in the cross-scale modeling framework.

### Utilizing relationships between crop growth and light interception

One of the first attempts to describe crop productivity, as opposed to leaf photosynthesis, as a function of radiation appeared in the work of de Wit (1959) and Loomis and Williams (1963). Many authors have since reported linear relationships between dry mass production and intercepted solar radiation for various species. The landmark paper by Monteith and Moss (1977) consolidated the grounds for this relationship both experimentally and theoretically (Sinclair and Muchow, 1999), and led to the term RUE. Calculation of canopy biomass growth with the RUE approach is achieved simply by multiplying solar radiation intercepted by the canopy (e.g., Equation 1), based on the canopy light distribution model (Monsi and Saeki, 1953), with a pre-determined RUE (Sinclair and Muchow, 1999). Although the RUE approach brings empiricism to the canopy level, as opposed to the leaf level with the PLR models, its simplicity and practicality facilitated its widespread adoption in crop modeling to quantify crop growth.

One of the first uses of the RUE approach in crop models appeared in Sinclair (1986) and it continues to have a significant role in more recent crop modeling advances (e.g., Hammer et al., 2010). The RUE is species dependent and is usually higher in C_4_ species than C_3_ species. Based on a total solar radiation basis (which is approximately double the photosynthetic active radiation), the RUE of maize under optimal growing conditions is 1.6–1.7 g/MJ, but can be as high as 1.9 g/MJ (Lindquist et al., 2005). Similarly, the RUE of pearl millet, which is another C_4_ species, is in the vicinity of 2.0 g/MJ (van Oosterom et al., 2002). For the C_4_ species sorghum, the RUE of 3-dwarf germplasm is generally only 1.2–1.4 g/MJ (Sinclair and Muchow, 1999), although a tall, 1-dwarf Indian hybrid had a RUE in the range of 1.6–1.8 g/MJ (Hammer et al., 2010), similar to maize and pearl millet. In contrast, for C_3_ species, the RUE of wheat is ~1.2 g/MJ, whereas soybean, which is a dicotyledonous legume crop with a different leaf structure, has a RUE of ~1 g/MJ (Sinclair and Muchow, 1999).

The RUE increases with the nitrogen status of the leaves, particularly if leaf N is limiting growth (Sinclair and Horie, 1989). For C_4_ crops maize and sorghum, RUE increases rapidly from a SLN level of 0.3 g/m^2^ and approaches a plateau when SLN reaches its critical value of ca. 1 g/m^2^ (Muchow and Sinclair, 1994), beyond which any increase in SLN has no significant effect on crop growth. For C_3_, rice the minimum SLN was similar to maize and wheat (0.3 g/m^2^), but the critical SLN was around 2 g/m^2^ (Sinclair and Horie, 1989). In general, C_3_ species tend to have greater SLN than C_4_ species (Anten et al., 1995). The vertical profile of SLN within a canopy also affects the RUE (Hammer and Wright, 1994), as can environmental factors such as the nature of incident solar radiation, air temperature (*T*_a_), atmospheric CO_2_ partial pressure (*C*_a_), and plant water status. The RUE increases with greater proportion of diffuse solar radiation (e.g., Sinclair et al., 1992), which is also predicted with a theoretical analysis that incorporates leaf-level photosynthesis light responses (Hammer and Wright, 1994). Their work predicted an increase of *ca* 40% in RUE of soybean crops (at SLN of 3 g/m^2^) with full diffuse solar radiation in case of heavy cloud cover.

Crop models commonly use simple indices as multipliers to capture effects of *T*_a_ and *C*_a_ and plant water status. For example, in APSIM-wheat it is assumed that RUE is not affected by temperature in the range of 10–25°C (http://www.apsim.info/Documentation/Model,CropandSoil/CropModuleDocumentation/Wheat.aspx), but is reduced at either higher or lower temperatures. In comparison, this range is 17–33°C in the CERES-Maize model (López-Cedrón et al., 2005). This insensitivity of RUE over broad ranges of temperatures corresponds with findings that leaf photosynthesis is insensitive to temperature within these ranges (Sage and Kubien, 2007). In modeling studies exploring impacts of climate change on crop productivity (Lobell et al., 2015), RUE is increased with elevated *C*_a_ in C_3_ species but is not changed in C_4_ species, in line with the known differences in response to photosynthesis. In water-limited situations, RUE is reduced in line with the relative transpiration achieved by the crop, which is determined from the balance between atmospheric demand and soil water uptake (Chapman et al., 1993). The RUE approach is a simpler way to model crop growth than the PLR models (used in the first type of canopy photosynthesis modeling), but it involves invoking a number of empiricisms to deal with responses to environmental factors and crop physiological attributes of the crop, which can reduce the predictive power of the RUE approach.

## Avenues to improve connections in the cross-scale modeling framework with environmental factors and crop physiological attributes

Success of cross-scale modeling depends on (1) the reliability of models at each level and (2) effective connections across levels of modeling. In previous sections, we have discussed the emerging framework for cross-scale modeling that connects photosynthesis models at the biochemical/leaf level with those at the canopy/crop level, with an emphasis on capture of light and its conversion into photosynthates and canopy biomass. Conveniently, this established framework can serve as the basis to connect other environmental factors and crop physiological attributes into the cross-scale model (Figure 1). Nitrogen is a critical factor in this connection and we will detail the use of SLN to exemplify the two considerations for cross-scale modeling: (1) generation and modeling of SLN as an emergent consequence of plant growth and development dynamics, and (2) developments for connecting SLN into the cross-scale modeling framework using the sun-shade leaf modeling approach.

### Crop physiological attribute: specific leaf nitrogen (SLN)

SLN is an emergent consequence of plant growth and development dynamics and has been modeled on this basis in crop models (Hammer et al., 2010). The rate of nitrogen (N) uptake of a crop is closely linked to leaf area expansion (Lemaire et al., 2007), particularly early in the crop cycle, prior to stem elongation (van Oosterom et al., 2010). Pre-anthesis allocation of N across organs follows a hierarchical pattern, where the demand for structural stem N and the N demand of expanding leaves is met first (Hammer et al., 2010). For leaves, this demand is represented by the critical SLN. Any additional N that is available once these demands have been met is subsequently allocated to these organs as luxury or storage N, which reflects the sink-limited crop growth modeling approach (Fatichi et al., 2014). Because larger stems require more structural N, stem size can reduce luxury leaf N uptake beyond the critical SLN. Similarly, a large canopy size (high LAI) will reduce SLN through dilution. As a consequence, the SLN of a crop is an emergent consequence of the complex interactions among total crop N uptake, relative organ size (which determines crop N demand) and the hierarchy of N allocation (van Oosterom et al., 2010).

SLN is a key driver of both crop-level RUE (e.g., Sinclair and Horie, 1989) and leaf-level photosynthesis (i.e., leaf CO_2_ assimilation rate) (e.g., Evans, 1989; Grindlay, 1997). Below the critical SLN, the rate of light-saturated (or CO_2_ limited) net photosynthesis increases linearly with SLN, although the response differs across species (Field and Mooney, 1983; Sinclair and Horie, 1989; Anten et al., 1995) and can also depend on environmental conditions. At SLN values above the critical level, however, photosynthesis rates reach a maximum (Field and Mooney, 1983; Sinclair and Horie, 1989), which is possibly linked to either light and/or the supply of CO_2_ being the limiting factor (Evans, 1989). One approach to modeling leaf-level photosynthesis that incorporates SLN and is adopted in some PLR models is to associate some key photosynthetic parameters with SLN (e.g., *A*_max_, Hammer and Wright, 1994). This approach is also applicable for driving the biochemical photosynthesis models with crop physiological attributes, which can be done by establishing relationships between biochemical photosynthesis model parameters (e.g., maximum rate of Rubisco carboxylation, *V*_cmax_) and SLN (e.g., de Pury and Farquhar, 1997). This approach allows SLN, which is often related to canopy-level RUE, to be connected with the leaf-level models, and thus facilitates effective connections across levels of modeling.

### Environmental factors: air temperature and CO_2_

Environmental factors such as air temperature (*T*_a_) and atmospheric CO_2_ partial pressure (*C*_a_) should be accounted for in the cross-scale modeling framework (Figure 1). Like solar radiation, *T*_a_ also varies diurnally and seasonally, which can also be modeled, for example, using the approach of Parton and Logan (1981). *T*_a_ influences leaf photosynthesis because it is a key determinant of leaf temperature (*T*_*l*_) (Monteith and Unsworth, 2013b).

One of the early studies of photosynthetic responses to *T*_*l*_ and *C*_a_ was on quantum efficiency of photosynthesis (Ehleringer and Björkman, 1977). Various studies quantified the effects of *C*_a_ on the empirical convexity factor of some PLR models (Ogren, 1993; Lewis et al., 1999) (attributed to the CO_2_ effect on photorespiration; Yin and Struik, 2015), and on the maximum rate of photosynthesis and quantum efficiency (Cannell and Thornley, 1998). In general, increases in *T*_*l*_ or *C*_a_ also increase values of some PLR model parameters. However, the combined effects of *T*_*l*_ and *C*_a_ on model parameters becomes more complex to analyse (Cannell and Thornley, 1998). The more aggregated nature of PLR models means that model parameters often represent a combination of biochemical processes of photosynthesis, each of which may respond differently to environmental effects. Crop models using the more aggregated forms of photosynthesis modeling (PLR or RUE) often incorporate scaling factors as multipliers to account for effects of temperature and *C*_a_ on growth (Jones et al., 2003; Holzworth et al., 2014; Lobell et al., 2015). Hence, the effects of *T*_*l*_ and *C*_a_ in crop models using these empirical formalisms cannot be estimated as reliably as with biochemical photosynthetic models.

## Connecting biochemical models of photosynthesis into the cross-scale modeling framework

An approach to reduce the empiricism at the biochemical/leaf level in cross-scale modeling involves using models that can better capture the biochemical processes of photosynthesis and, at the same time, ensures effective connections across levels of modeling. This was pioneered by Yin and van Laar (2005), who connected the biochemical models of photosynthesis with the GECROS crop model through upscaling to canopy photosynthesis with the sun-shade leaf modeling approach. This cross-scale modeling approach was used by Yin and Struik (2008) to simulate likely consequences on rice yield of introducing the C_4_ pathway into rice. In a later study, Gu et al. (2014) demonstrated that the cross-scale model could be used to explore effects of natural variation in photosynthetic attributes on biomass accumulation of rice. Yin and Struik (2015) have utilized the cross-scale modeling framework and evaluated constraints to the potential efficiency of converting solar energy into phytoenergy along the scales of biological organization from leaf biochemistry to canopy physiology and crop biomass. However, the latter two studies stopped short of exploring effects on grain yield because of uncertainties related to the prediction of grain number. The choice of models at both the biochemical/leaf and canopy/crop level are equally important for bridging the gap between photosynthetic manipulation at the biochemical level and crop productivity. Another canopy photosynthesis modeling study, which was also developed through upscaling of biochemical models, was undertaken to explore consequences of changing Rubisco kinetic properties on daily canopy photosynthesis (Zhu et al., 2004; Long et al., 2006). This study, however, was limited to daily predictions, because the simulation model lacked the two-way connection between the biochemical models and crop growth and development dynamics. In the following section, we will discuss strategies for connecting the biochemical models of photosynthesis into the cross-scale modeling framework to overcome issues of connectivity.

### Biochemical models of C_3_ and C_4_ leaf photosynthesis

The importance of Ribulose-1,5-bisphosphate carboxylase/oxygenase (Rubisco) in determining photosynthesis has been recognized in early studies (see overview in von Caemmerer, 2013). Rubisco catalyses the competing reactions of the carboxylation [first step of the photosynthetic carbon reduction (PCR) cycle] and oxygenation [first step of the photorespiratory carbon oxidation (PCO) cycle] of ribulose-1,5-bisphosphate (RuBP) (Figure 1). This led to the development of biochemical photosynthesis models based on Rubisco kinetic properties and the two cycles (see review of von Caemmerer et al., 2009). The model of Farquhar et al. (1980) is one of these, and will be referred to here as the C_3_ photosynthesis model. This model assumes that net photosynthesis is determined by the minimum (Equation 2) of either RuBP-saturated (Rubisco-limited) or RuBP-regeneration-limited (electron-transport-limited) CO_2_ assimilation rate (Equations 3, 4 respectively). Rubisco-limited photosynthesis is comparable to the CO_2_-limited photosynthesis described by Blackman's PLR model, whereas electron-transport-limited photosynthesis is comparable to the light-limited photosynthesis. Farquhar (1989) demonstrated that the C_3_ photosynthesis model, intended at the biochemical level, is also applicable for photosynthesis at the leaf level given the assumptions that photosynthetic attributes are identical for all chloroplasts in the leaf and that light distribution inside the leaf is homogenous (Song et al., 2013). The utility of the C_3_ photosynthesis model has been in helping to interpret gas exchange measurements of steady-state CO_2_ assimilation (*A*) of leaves (e.g., response of *A* to intercellular CO_2_ partial pressure, *A*/*C*_i_ curves) and predicting the effects on photosynthesis of variation in genotype, photosynthetic photon flux density (PPFD), leaf temperature, and intercellular CO_2_ and O_2_ partial pressures (von Caemmerer et al., 2009). The main equations of the C_3_ photosynthesis model (von Caemmerer, 2000) are:

(2) $$\begin{array}{l} A=\text{min}\left\{A_{\text{c}},A_{\text{i}}\right\} \end{array}$$

(3) $$\begin{array}{l} A_{\text{c}}=\frac{\left(C_{\text{c}}\;-\;\Gamma_{*}\right)V_{\text{cmax}}}{C_{\text{c}}\;+\;K_{\text{c}}\left(1\;+\;{\text{O}}_{\text{c}}/K_{\text{o}}\right)}-R_{\text{d}} \end{array}$$

(4) $$\begin{array}{l} A_{\text{j}}=\frac{\left(C_{\text{c}}\;-\;\Gamma_{*}\right)J}{4C_{\text{c}}\;+\;8\Gamma_{*}}-R_{\text{d}} \end{array}$$

where *A* is the net CO_2_ assimilation rate, *C*_c_ and *O*_c_ are the chloroplastic CO_2_ and O_2_ partial pressures respectively, *R*_d_ is the respiration other than that from the PCO cycle, Γ_*_ is half the reciprocal of the relative CO_2_/O_2_ specificity of Rubisco multiplied by *O*_c_ and is also defined by the CO_2_ compensation point in the absence of *R*_d_ (Figure 2.11, von Caemmerer, 2000), *V*_cmax_ is the maximum rate of Rubisco carboxylation, *K*_c_ and *K*_o_ are the Michaelis Menten constants of Rubisco carboxylation and oxygenation, and *J* is the electron transport rate.

The photosynthetic electron transport chain can be limited by either NADPH (the reduced form of nicotinamide adenine dinucleotide phosphate) or ATP (adenosine triphosphate) supply, depending on which of the three modes of electron transport (i.e., linear, cyclic, and pseudocyclic electron transport) are active (von Caemmerer, 2000). Equation (4), the most used expression, assumes 100% linear electron transport and that NADHP supply limits overall leaf photosynthesis (Yin et al., 2009). However, this may not always be the case in leaves. For example, if ATP supply is limiting with 100% linear electron transport, the factors 4 and 8 in the denominator of Equation (4) are replaced by 4.5 and 10.5, respectively (Yin et al., 2009). Due to the uncertainty on whether NADPH or ATP is limiting, different forms of Equation (4) are used. This uncertainty can be eliminated by using a generalized stoichiometry of the electron transport chain (extended-electron-transport-chain) model that incorporates the three modes of electron transport for C_3_ photosynthesis (Yin et al., 2009). A C_4_ equivalent of the model is presented in Yin and Struik (2009). The extended model quantifies the photosynthetic quantum yield (α) based on the photochemical efficiencies of photosystem I and II and the fraction of total photosystem I electron fluxes that follows the cyclic and pseudocyclic pathways (Yin and Struik, 2015). The extended model is valuable for the analysis of photosynthetic regulation via the electron transport pathways in response to environmental stresses (Yin et al., 2004, 2009). However, there are still uncertainties in a number of parameters in the model (e.g., number of H^+^ transported by cyclic electron transport; Yin and Struik, 2009). In addition, Equation (4) performed similarly to the extended-electron-transport-chain model when used to infer values of *V*_camx_ and *J*_max_ of the C_3_ photosynthesis model from *A*/*C*_i_ curves (Yin et al., 2004). In fact, this is also the case for the ATP-supply-limited version of Equation (4). This implies that for the purpose of reproducing *A*/*C*_i_ curves for cross-scale modeling, use of the extended-electron-transport-chain model may not be necessary.

The main equations for the biochemical model of C_4_ photosynthesis (von Caemmerer, 2000), based on previous work of Berry and Farquhar (1978) and Collatz et al. (1992), adopts the main equations of the C_3_ photosynthesis model (i.e., Equations 2–4) with modifications to capture the coordinated function of mesophyll and bundle-sheath cells. In the C_4_ photosynthesis model, *A*_c_ and *A*_j_ are given by:

(5) $$\begin{array}{l} A_{\text{c}}=\frac{\left(C_{\text{s}}\;-\;\gamma_{*}O_{\text{s}}\right)V_{\text{cmax}}}{C_{\text{s}}\;+\;K_{\text{c}}\left(1\;+\;O_{\text{s}}/K_{\text{o}}\right)}-R_{\text{d}} \end{array}$$

(6) $$\begin{array}{l} A_{\text{j}}=\frac{\left(C_{\text{s}}\;-\;\gamma_{*}O_{\text{s}}\right)\left(1\;-\;x\right)J_{\text{t}}}{3\left(C_{\text{s}}\;+\;7/3\gamma_{*}O_{\text{s}}\right)}\;-\;R_{\text{d}} \end{array}$$

where γ_*_ is half the reciprocal of the relative CO_2_/O_2_ specificity of Rubisco, *C*_s_ and *O*_s_ are the bundle-sheath CO_2_ and O_2_ partial pressure [which are calculated with additional equations, described in von Caemmerer (2000), reflecting the coordinated function of mesophyll and bundle-sheath cells], respectively. *J*_t_ is the total electron transport rate from both the mesophyll and bundle-sheath cells and the factor (1 − *x*) represents the proportion of the total electrons that are available to the bundle-sheath cells, while the rest are required by the mesophyll cells.

### Connecting biochemical photosynthesis models to environmental factors and crop physiological attributes

Biochemical photosynthesis models can be connected to crop models by utilizing the emerging cross-scale modeling framework (Figure 1). The first step in this approach, which involves upscaling to canopy photosynthesis, has been achieved through the use of the simpler representations of canopy structure (i.e., LAI and *k*) such as the multi-layer (Leuning et al., 1995), the sun-shade (de Pury and Farquhar, 1997), the big-leaf (which aggregates absorbed radiation and photosynthetic capacity into a single element) (Sellers et al., 1992) modeling approaches and 3D models such as the RATP model (Sinoquet et al., 2001). However, upscaling to cross-scale modeling through the use of the sun-shade modeling approach is an avenue to achieve a balance between simplicity and robustness (Yin and van Laar, 2005). In the following section, we will discuss strategies for connecting environmental factors (i.e., *T*_a_, *C*_a_, and water) and crop physiological attributes (e.g., SLN) into the cross-scale modeling framework.

An issue for canopy photosynthesis modeling is if and how to include spatial variability within the canopy for environmental factors like *T*_a_ and *C*_a_. Many authors resort to the assumption that these environmental factors do not vary spatially within the canopy (Leuning et al., 1995; Wang and Leuning, 1998; Yin and van Laar, 2005), while others use empirical functions, such as an exponentially decreasing wind speed through canopy for estimating leaf boundary layer conductance, which affects diffusion of *C*_a_ into leaves (Yin and van Laar, 2005). Spatial variability of environmental parameters within the canopy, such as *T*_a_, can also be captured with 3D plant architecture modeling (e.g., Sinoquet et al., 2001). However, the assumptions for omitting spatial variability are supported by observations (Wang and Leuning, 1998) that the effects on canopy photosynthesis of including spatial variation for *T*_a_, vapor pressure deficit (VPD) and *C*_a_ in simulations are only minor (within 5%) under a wide range of soil water availabilities and meteorological conditions. This is further supported by the finding that canopy photosynthesis can be closely approximated by photosynthesis of the sunlit fraction (Hirose, 2005). It could be that a large part of canopy photosynthesis is contributed by leaves in the upper part of the canopy, which experience environmental conditions close to those above the canopy, making it reasonable to omit the spatial variability in *T*_a_, VPD, and *C*_a_ within canopies from simulation models.

As seen in the Section Utilizing Relationships between Crop Growth and Light Interception, *T*_a_ can have a significant impact on RUE and crop growth, because it affects leaf temperature (*T*_l_), which in turn affects leaf-level photosynthesis. The question of how to estimate *T*_l_ is covered in subsequent paragraphs. First, parameterizing effects of *T*_l_ on photosynthesis is possible by using studies that quantify the response of biochemical model parameters to *T*_l_ (Bernacchi et al., 2001, 2002, 2003; Massad et al., 2007; Braune et al., 2009; Boyd et al., 2015; Yin et al., 2016). Temperature responses of the main C_3_ model parameters (*V*_cmax_, *J*_max_, *K*_c_, *K*_o_, and Γ_*_) have been studied both *in vivo* and *in vitro* in recent years. *In vivo* data sets are most comprehensive for model C_3_ species such as *Arabidopsis thaliana* and *Nicotiana tabacum* (Bernacchi et al., 2001, 2002, 2003; Walker et al., 2013), although data sets for C_3_ and C_4_ crop species are also becoming available (Massad et al., 2007; Braune et al., 2009; Yin et al., 2016). *In vitro* data for C_3_ species like wheat (Cousins et al., 2010) and the model C_4_ species *Setaria viridis* have also appeared (Boyd et al., 2015). To connect *T*_l_ into canopy photosynthesis modeling, it has been suggested that spatial variation of *T*_l_ within the canopy is required. 3D canopy architectural models could be used to describe this spatial variation (Sinoquet et al., 2001). However, Wang and Leuning (1998) demonstrated by using the sun-shaded leaf modeling approach, assuming that *T*_l_ was the same within each fraction but differed between them, that a reasonable approximation resulted. This has been implemented in many studies (de Pury and Farquhar, 1997; Wang and Leuning, 1998; Dai et al., 2004; Yin and van Laar, 2005).

The difficulty in simulating *T*_l_ is that it is interlinked with transpiration. Diffusion of *C*_a_ into leaves through stomata (*C*_i_) driving photosynthesis is affected by stomatal conductance, which is driven by the balance between atmospheric demand and crop soil water uptake. These complex interactions are unavoidable. An emerging framework for capturing these interactions in well-watered situations is by coupling the leaf-level biochemical photosynthesis models with the isothermal form of the Penman-Monteith equation (for *T*_l_ simulation) and a stomatal conductance model (for *C*_i_ simulation). The Penman-Monteith equation (Monteith, 1965a; Monteith and Unsworth, 2013b) reflects the interdependency between leaf energy balance and transpiration, which depends on diffusion of water vapor out of leaves through stomata and the leaf boundary layer and on the VPD of the surrounding air. Many authors have taken this path and captured these interactions for leaf-photosynthesis modeling and connected it with canopy photosynthesis modeling through the use of the sun-shade modeling approach (Wang and Leuning, 1998; Kim and Lieth, 2003; Yin and van Laar, 2005; Yin and Struik, 2009). Furthermore, some of these canopy photosynthesis models have been implemented in crop models (Yin and van Laar, 2005; Yang et al., 2009), achieving frameworks in line with the cross-scale modeling approach. Given this framework for capturing connections across scales, there remain choices of models for capturing the interlinked nature of *T*_l_, transpiration, and photosynthesis.

Canopy photosynthesis models that respond to *C*_a_, which affects *C*_i_, involve incorporating a stomatal conductance model. The most widely used leaf stomatal conductance models are those that relate stomatal conductance to photosynthesis (e.g., the BWB-type models developed and named after the authors in Ball et al., 1987) and multiplicative models (e.g., Jarvis, 1976) based on environmental factors (for review of stomatal conductance models see Damour et al., 2010). Stomatal conductance for CO_2_ can be converted to that for water vapor, or *vice versa*, by assuming water vapor diffuses through stomata at a rate that is 1.6 times faster than CO_2_ (Dai et al., 2004; Yin and van Laar, 2005). This is modeled by using Graham's law of effusion, calculated as the square root of the ratio of the molecular weight of CO_2_ over H_2_O. The BWB-type models have subsequently been improved by several authors (Leuning, 1995; Yin and Struik, 2009), while retaining the concept that stomatal conductance is driven by photosynthesis. Coupling of stomatal conductance models to biochemical models of photosynthesis (Collatz et al., 1992; Leuning, 1995; Kim and Lieth, 2003; Yin and Struik, 2009; Li et al., 2012) can facilitate photosynthesis estimation in response to *C*_a_. Wang and Leuning (1998) incorporated a BWB-type stomatal conductance model into canopy photosynthesis modeling through the use of the sun-shade modeling approach. They assumed that stomatal conductance (per leaf area basis) was the same within each fraction but differed between them, and concluded that this generated a reasonable approximation. This is a simple and robust approach for connecting *C*_a_ into canopy photosynthesis modeling and has been adopted in other work (Dai et al., 2004; Yin and van Laar, 2005).

The emerging framework for capturing the interactions between *T*_l_, transpiration and photosynthesis is subsequently revised to capture effects of water-limited situations. Many authors incorporated empirical effects on some of the biochemical model parameters (see references cited in Li et al., 2012), assuming that water stress directly affects photosynthesis. In contrast, Li et al. (2012) modeled effects of water stress by incorporating effects of soil water as an empirical impact function in the BWB-type model without modifying photosynthesis. This can be upscaled to the canopy level as described above. However, the similarity in both approaches to incorporating water effects is that stomatal conductance is responsive to photosynthesis, which may not be the case in water-limited situations. Approaches to water limitation used in crop models may provide an avenue to reduce the empirical approach currently used in biochemical photosynthesis models. When water becomes limiting, some crop models drive crop growth via transpiration, which is estimated by the balance between atmospheric demand and crop soil water uptake (Monteith and Greenwood, 1986; Monteith, 1988; Hammer et al., 2010). The switch between light-limited and water-limited crop growth depends on the estimated plant water status (Chapman et al., 1993; Hammer et al., 2010). A more mechanistic method involving the coordination of the controls of stomatal aperture, transpiration and abscisic acid (Tardieu et al., 2015) can be used to estimate transpiration, but the simpler approach for estimating transpiration in crop models has proven to be robust when applied across a diverse range of environments (Hammer et al., 2010). This implies that in water-limited situations, photosynthesis is responsive to stomatal conductance.

A major crop physiological attribute affecting canopy and leaf photosynthesis is SLN (Section Avenues to Improve Connections in the Cross-Scale Modeling Framework with Environmental Factors and Crop Physiological Attributes). Nitrogen is needed to support photosynthetic machineries via Rubisco for the photosynthetic carbon reduction cycle and thylakoid membrane proteins for the electron transport chain. Partitioning of leaf nitrogen to these photosynthetic components is a complex topic and is probably species dependent (Evans, 1989; Buckley et al., 2013). Fortunately, without modeling these complexities, canopy photosynthesis can be linked to canopy nitrogen content through upscaled PLR models that respond to SLN (Hammer and Wright, 1994). This approach is also applicable for obtaining canopy photosynthesis based on the biochemical photosynthesis models (de Pury and Farquhar, 1997). Linking biochemical photosynthesis model parameters to SLN becomes a key for connecting across scales, because model parameters are quantitatively related to SLN (Evans, 1983, 1989; Braune et al., 2009; Archontoulis et al., 2012). Because of this association, total nitrogen content of sunlit and shaded fractions can be used to drive the biochemical photosynthesis models when scaling up to the canopy level (de Pury and Farquhar, 1997).

### Extending cross-scale modeling for assessing genetic manipulations of photosynthesis

Current canopy photosynthesis modeling based on the biochemical photosynthesis models can be readily used for assessing the consequences of genetic manipulations of photosynthesis on crop growth and production. Yin and Struik (2008) incorporated a canopy photosynthesis model, based on the C_4_ photosynthesis model, into the GECROS crop model to assess the consequences of C_4_ photosynthesis on rice crop production. They predicted a yield increase of 23% with the full introduction of the C_4_ pathway into rice. Improvement in Rubisco kinetic properties have also been assessed for consequences on canopy photosynthesis (Zhu et al., 2004; Long et al., 2006). Given the wide range of possibilities for photosynthetic manipulation there is a need to extend the biochemical models and possibly the cross-scale modeling framework. Three important areas in pursing this are (1) extending the biochemical photosynthesis models to capture genetic manipulations in photosynthesis, (2) connecting model extensions across scales, and (3) capturing pleiotropic effects of genetic manipulations in photosynthesis. These issues are discussed in terms of framing the cross-scale modeling for genetic manipulations using examples based on current knowledge for three specific potential manipulations.

#### Improving Rubisco kinetic properties

The upscaled biochemical photosynthesis models (Section Connecting Biochemical Photosynthesis Models to Environmental Factors and Crop Physiological Attributes) can be readily used to estimate consequences of manipulations in Rubisco kinetics on canopy photosynthesis. However, an effect of changing Rubisco kinetics, *K*_c_ in particular, is that it can also affect the maximum catalytic turnover of carboxylase per Rubisco site, *k*_cat_ (Whitney et al., 2011; Evans, 2013; Sharwood and Whitney, 2014). In addition, *V*_cmax_ is the product of *k*_cat_ and the number of moles of Rubisco sites per unit of leaf area, *n*_R_ (Zhu et al., 2004; Evans, 2013). These pleiotropic effects will affect the calculation of CO_2_ assimilation rate and should be captured in the cross-scale modeling. However, *n*_R_ and the relationship between *K*_c_ and *k*_cat_ need to be defined. *n*_R_ can be calculated from measurements of Rubisco concentration in leaves, which provides the connection with SLN, which in turn is determined by the balance between crop nitrogen uptake and rate of leaf area growth [Section Crop Physiological Attribute: Specific Leaf Nitrogen (SLN)]. However, the relationship between *K*_c_ and *k*_cat_ may be difficult to predict as different approaches for manipulating Rubisco enzymes can lead to different relationships (Evans, 2013). On the other hand, when comparing *K*_c_ and *k*_cat_ of Rubisco enzymes, there is a strong linear relationship (Evans, 2013) that can be parameterized by a slope and intercept. Capturing these pleiotropic effects in the cross-scale modeling should allow more realistic simulations of canopy photosynthesis.

#### Mesophyll conductance

The biochemical photosynthesis models have already been extended to include diffusion of *C*_a_ from the surrounding air to the leaf intercellular airspace (*C*_i_) by incorporating the leaf boundary layer conductance and stomatal conductance and have been incorporated into canopy photosynthesis models (Wang and Leuning, 1998). However, mesophyll conductance, which affects diffusion of *C*_i_ to the carboxylating site of Rubisco (*C*_c_), is also required in modeling due to its significance in controlling leaf photosynthesis (Flexas et al., 2008; Terashima et al., 2011) and for considering mesophyll conductance manipulations (Flexas et al., 2012). The framework to include mesophyll conductance in modeling CO_2_ diffusion, using Ficks's first law of diffusion for CO_2_, is given by:

(7) $$\begin{array}{l} C_{\text{c}}=C_{\text{a}}-\frac{A}{g_{\text{bl}}}-\frac{A}{g_{\text{s}}}-\frac{A}{g_{\text{m}}} \end{array}$$

where *C*_c_ and *C*_a_ are the CO_2_ partial pressure at the carboxylating site of Rubisco inside chloroplasts and of the surrounding air, respectively, *g*_bl_, *g*_s_ and *g*_m_ are the leaf boundary layer, stomatal and mesophyll conductance for CO_2_ respectively, and *A* is the net CO_2_ assimilation rate.

Use of Equation (7) relies on revising *C*_i_-based biochemical model parameters to incorporate effects of *g*_m_ (i.e., *C*_c_-based values) and parameterizing of *g*_m_. The first issue is accommodated by the increasing availability of *C*_c_-based values (Bernacchi et al., 2002; Cousins et al., 2010). For the second issue, environmental factors (e.g., air temperature, soil water, and *C*_a_), crop physiological attributes (e.g., leaf N), and leaf anatomical parameters (e.g., mesophyll cell wall thickness) can be used to determine *g*_m_ through the use of empirical relationships (Evans et al., 2009; Flexas et al., 2012; Tomás et al., 2013). These empirical approaches rely on experimental measurements for establishing the correlation, which can be facilitated by developments in measurement techniques such as gas exchange, in combination with carbon isotope discrimination (Pons et al., 2009). Such techniques allow the response of *g*_m_ to soil N, soil water, and leaf temperature to be parameterized (Evans and von Caemmerer, 2013; Barbour and Kaiser, 2016). Established correlations with empirical approaches, however, may not extrapolate reliably to yet unexplored parts of the simulation landscape. For example, *g*_m_ responses to environmental factors and crop physiological attributes may not be estimated using known empirical relationships when *g*_m_ is manipulated by altering expression of aquaporins on cell membranes (Flexas et al., 2012) or anatomical features of leaves such as the thickness of mesophyll cell walls (Terashima et al., 2011). An additional layer of complexity is introduced if pleiotropic effects of *g*_m_ manipulation are considered. Work on characterizing effects of *g*_m_ manipulation on a range of photosynthesis related attributes (e.g., total Rubisco activity, respiration rate, and *g*_s_) can provide information on possible pleiotropic effects (Flexas et al., 2006; Barbour et al., 2016), which should help parameterize the cross-scale modeling. Recently, a more mechanistic approach for modeling *g*_m_ was presented (Evans and von Caemmerer, 2013). However, it should be noted that use of more complex models will need parameterization of responses to environmental and crop physiological attributes for the cross-scale modeling. Effective modeling involves careful consideration of the extent of complexity actually required (Hammer et al., 2006).

#### Introducing chlorophyll *d* and *f* into the light harvesting antenna of photosystems

Light absorption by Chlorophyll *a* and *b*, which are the most common chlorophylls in the light harvesting antenna of crop species, can only utilize wavebands in the spectrum up to 700 nm (Chen and Blankenship, 2011). Introducing chlorophyll *d* and *f* could offer the possibility of pushing the boundary toward 750 nm, which increases available PAR by 19% (Chen and Blankenship, 2011). Effects of this can be estimated by existing canopy light distribution models (e.g., sun-shade models) if *k*-values can be revised. However, the biochemical photosynthesis models commonly use an empirical, non-rectangular hyperbola function to describe electron transport rate (*J*) in terms of PPFD (e.g., Equation 2.13, von Caemmerer, 2000). Unfortunately, the connection between empirical parameters of the non-rectangular hyperbola function and compositions of chlorophyll types is not clear. In addition, these new chlorophyll types will alter the spectral quality of light in the canopy by changing the ratio between red (660 nm) and far-red (730 nm) light. This could interfere with the phytochromes in plants that regulate shade avoidance and other phytochrome-related physiological functions (Evans, 2013). It has been shown that lower red:far-red ratio can cause a lower chlorophyll *a*:*b* ratio (Pons and de Jong-van Berkel, 2004) as well as influence bud outgrowth and tillering (Ballaré and Casal, 2000; Lafarge et al., 2002). These possible pleiotropic effects of introducing new chlorophyll types remain difficult to incorporate at this stage.

## Concluding remarks

The emerging cross-scale modeling framework connecting biochemical/leaf and canopy/crop levels (Figure 1) has the capacity to link genetic manipulation of photosynthesis to crop yield. The success of cross-scale modeling is built on (1) the reliability of models at each scale and (2) how well the connections are captured across scales. Connecting the biochemical photosynthesis models into the cross-scale modeling framework allows clearer links to the biochemical processes of photosynthesis, based on which consequences of photosynthetic manipulations can be reliably estimated. This can be further improved by the advancing understanding of photosynthetic responses to environmental and physiological attributes and further modeling efforts. The crop scale models with the concept of both source- and sink-limited growth provide important effects that can regulate the biochemical photosynthetic models via crop scale nitrogen and water status, which determine SLN and influence canopy conductance in water-limited situations. Development of the cross-scale modeling framework using the gene-to-phenotype modeling approach can potentially accelerate progress in improving crop resource capture efficiency to support crop improvement through genetic manipulation of photosynthesis.

## Author contributions

GH took part in securing the funding. AW, YS, EvO, and GH contributed substantially to the conception of this work, AW and YS drafted the work with help of EvO and GH. AW, EvO, and GH critically revised the work.

## Funding

The work is financially supported by the Australian Research Council through the Centre of Excellence for Translational Photosynthesis.

### Conflict of interest statement

The authors declare that the research was conducted in the absence of any commercial or financial relationships that could be construed as a potential conflict of interest.
